# Use of ^1^H-nuclear magnetic resonance to screen a set of biomarkers for monitoring metabolic disturbances in severe burn patients

**DOI:** 10.1186/cc13999

**Published:** 2014-07-24

**Authors:** Yong Zhang, Bin Cai, Hua Jiang, Hong Yan, Hao Yang, Jin Peng, Wenyuan Wang, Siyuan Ma, Xiuwen Wu, Xi Peng

**Affiliations:** State Key Laboratory of Trauma, Burns and Combined Injury, Institute of Burns of PLA, Southwest Hospital, Third Military Medical University, Chongqing, 400038 People’s Republic of China; Department of Computational Mathematics and Biostatistics, Metabolomics and Multidisciplinary Laboratory for Trauma Research, Sichuan Provincial People’s Hospital, Sichuan Academy of Medical Sciences, No. 585, Da Mian Hong He Bei Lu, Chengdu, Sichuan Province 610101 China

## Abstract

**Introduction:**

To establish a plasma metabolomics fingerprint spectrum for severe burn patients and to use it to identify a set of biomarkers that could be used for clinical monitoring.

**Methods:**

Twenty-one severe burn patients and three healthy control individuals were enrolled in this study, and the plasma samples from patients and healthy individuals were collected for nuclear magnetic resonance (NMR) measurements. The NMR spectra were analyzed using principal component analysis (PCA) and partial least squares (PLS) in order to establish the metabolomics fingerprint representing the changes in metabolism and to select the major biomarkers.

**Results:**

NMR spectra of the plasma samples showed significant differences between burn patients and healthy individuals. Using metabolomics techniques, we found an Eigen-metabolome that consists of 12 metabolites, which are regulated by 103 enzymes in a global metabolic network. Among these metabolites, α-ketoisovaleric acid, 3-methylhistidine, and β-hydroxybutyric acid were the most important biomarkers that were significantly increased during the early stage of burn injury. These results suggest that the mitochondrial damage and carbohydrate, protein and fatty acid metabolism disturbances occur after burn injury. Our analysis also show that histone deacetylases, which are protein transcription suppressors, were remarkably increased and indicate that protein transcription was inhibited and anabolism was restrained during the early stage of burn injury.

**Conclusions:**

Metabolomics techniques based on NMR can be used to monitor metabolism in severe burn patients. Our study demonstrates that integrated ^1^H-NMR metabolome and global metabolic network analysis is useful for visualizing complex metabolic disturbances after severe burn injury and may provide a new quantitative injury severity evaluation for future clinical use.

**Trial registration:**

Chinese Clinical Trial Registry ChiCTR-OCC-12002145. Registered 25 April 2012.

## Introduction

Burn is a common injury with an incidence of about 0.2% in the normal population. Every year, approximately 3 million people in China and 0.8 million in the United States suffer from burns, with 200,000 and 40,000 requiring hospitalization, respectively [1, 2]. In addition, more than one-third of burn patients are children under 14 years of age [1, 3]. Therefore, the treatment course for burns is not only a public healthcare issue, but also a relevant matter in the growth and future of children.

Mild burn is easy to treat, and the cure rate is 95% or greater worldwide. However, severe burn, which covers more than 50% of the total body surface area (TBSA), is very difficult to treat, and the mortality rate is usually more than 30%. Among the extremely severe burn patients for whom more than 80% of the TBSA is burned, the death rate can reach 70% or higher [1]. Although much research has been done and numerous advances have been made through the hard work of a generation of burn surgeons and scientists, the mortality of severe burn patients has not changed in the past decade [4–6]. Determining how to reduce the mortality and improve the care of severe burn patients is a core issue in burn research. After severe burn injury, along with massive damage to the skin and subcutaneous tissue, multiple organs are also damaged. Pathophysiological conditions are complicated and are highly related to metabolic regulation [7–9]. Therefore, understanding the complicated changes in metabolic networks is essential for developing the next generation of prognosis prediction tools and new treatment methods. However, metabolic regulatory networks involve large numbers of molecules and pathways. Conventional laboratory testing only includes a few of metabolic parameters and cannot measure global changes in metabolic networks in real-time. A metabolomics test based on ^1^H-nuclear magnetic resonance (NMR) provides a unique high-throughput solution to resolve this challenge. It can be used to detect most small metabolic molecules in a single-use test [10–12]. By using advanced mathematical modeling, researchers can visualize the global changes in metabolic networks (metabolic profile or metabolome) and extract a set of biomarkers. These biomarkers offer a new approach to quantitative, real-time monitoring for severe burn patients and would give clinical practitioners new opportunities to make better informed decisions.

One of the major challenges in analyzing NMR data from plasma samples is the high-dimension disaster of metadata. The only solution to address this challenge is to use a pattern recognizing technique. Principal component analysis (PCA) and partial least square (PLS) are two common algorithms that can be used for dimension reduction in NMR data analysis. Compared to PCA, PLS considers correlations between variables. Hence, both PCA and PLS are used as conventional mathematical tools in NMR data analysis. In our previous studies, we successfully used PCA and PLS to fit data according to the severity of spinal cord injury [13, 14]. We have reasonable confidence that these algorithms can be used to establish a metabolomic profile for severe burn patients, who suffer much greater metabolic disturbances. In addition, with the release of the Human Metabolome Database (HMDB), matching peaks to metabolites is now becoming much easier than before [15]. In brief, after peaks are screened using PCA and PLS, we can submit these peaks to HMDB and identify related metabolites. In the present study, by using a high-resolution NMR technique, we aimed to establish a plasma metabolomics fingerprint spectrum of severe burn patients and to use it to identify a set of biomarkers that can be used for clinical monitoring and to better understand metabolism disturbances after burns. With this effort, we expect to lay a foundation for formulating reasonable improvements to future treatment protocols.

## Materials and methods

### Subjects

Subjects were 21 adult severe burn patients admitted to the Institute of Burn Research of the Southwest Hospital of The Third Military Medical University between May 2012 and December 2012. Patients were recruited if they met the inclusion criteria of being between 18 and 65 years of age and having a burn area covering more than 50% TBSA. The exclusion criteria were: (1) special burns including chemical and electrical burns; (2) severe complications such as heart disease, hepatic disease, renal disease, and hematopoietic disease before burn; (3) oncologic disease; (4) history of endocrine disease including diabetes and hyperthyroidism; (5) obesity (body mass index >25 kg/m^2^); (6) pregnancy or lactation; (7) psychiatric disorder or mental state leading to failure to cooperate, inability for self-control, or trouble communicating; and (8) participation in other clinical trials.

Written informed consent was obtained from all participants, and the Committee of Medical Ethics of the Southwest Hospital of The Third Military Medical University approved the study protocol (approval number: KY201118).

### Clinical course of severe burn patients

All patients were admitted 2 to 24 hours post burn. When a patient was admitted, we applied the standardized fluid resuscitation protocol according to the Chinese Medical Association burn treatment guidelines to treat burn shock immediately. Silver sulfadiazine was applied to the wound, and systemic antibiotics were used to prevent infection. Escharectomy and skin grafting were performed three days after burn and three to four times within one month to help cover the wound surface.

### Collection and preparation of blood samples

Healthy controls were kept off food and water before blood collection at 8 a.m. Two milliliters of blood was collected from the median cubital vein using a citrate vacuum tube. The samples were centrifuged at 3,000 rpm for 10 minutes immediately, after which 1 ml of supernatant plasma was extracted. The supernatant was stored at -80°C until analysis. For severe burn patients, fasting blood samples were collected at 8 a.m. on the first morning after admission (24 to 48 hours post burn) and then processed as described for the controls.

Plasma samples were defrosted at room temperature and centrifuged at 16,000 rpm for 10 minutes. Then 450 μl supernatant was extracted from each sample and fully mixed with 50 μl deuterium oxide (D_2_O) for 120 seconds. After standing for 10 minutes, samples were analyzed using 600-MHz NMR spectrometry.

### NMR measurements and data analysis

#### NMR measurements

We employed NMR measurements according to a protocol that was established and reported previously [13]. All one-dimensional spectra were acquired at 600.13 MHz using a Bruker Avance DRx 600 600-MHz spectrometer (Bruker BioSpin GmbH, Rheinstetten, Germany) equipped with a proton observation probe (Bruker BBI inverse-broadband probe). Spectra were recorded at a room temperature of 300 K. Standard one-dimensional pulse sequences and Carr-Purcell-Meiboom-Gill (CPMG) sequences were used. A spin-spin relaxation delay of 64 ms was used for all samples, and water suppression irradiation was applied during the relaxation delay (2 s). Typically, in the standard one-dimensional and CPMG experiments, the spectral width was 20 ppm and 256 transients were collected into 32 k data points. CPMG experiments filter broad resonances from proteins and lipids, permitting latent biomarkers of smaller molecular weight to be visualized.

#### Data processing

Clinical data were described as mean ± standard deviation (SD) or as median and interquartile range (IQR) in the case of a skewed distribution. Differences between groups were assessed with the Student’s *t* test for data presented as means. Differences in counts or percentages were evaluated with the Fisher’s exact probability test. Differences were considered significant if a two-tailed *P* value was <0.05.

All plasma ^1^H-NMR spectra were phased and baseline corrected within mestReC (version 4.9.9.9, Mestrelab Research SL, Rheinstetten, Germany), and the chemical shifts were referenced to a creatinine peak at Δ3.05. These data were introduced into a Matlab (R2012b, The MathWorks, Inc, Natick, MA, USA) data structure, where each row comprised the integral descriptors for an NMR spectrum. To reduce the interference of huge water peaks, all spectra were analyzed to non-normalized data after removal of the spectral region containing the suppressed water resonance.

#### Pattern recognition

All multivariate statistics and pattern recognition were performed using the Eigen victor toolbox (ver6.2.1) with two techniques: PCA and PLS on the Matlab. Before analyzing, scaling was applied to minimize the variation of the ^1^H-NMR peak to ensure that the large peak did not overshadow the contribution of the small one. PCA score plots were constructed to visualize the inherent clustering of the samples based on burning. The toolbox can export the Q^2^ value, which indicates how well the model predicts new data. A large Q^2^ (>0.5) indicates good predictive capability.

For further analysis, PLS-discriminant analysis (PLS-DA) was used in the data processing. PLS is used to find the fundamental relationship between two matrices (X and Y), that is, a latent variable approach to modeling the covariance structures in these two spaces. Here the X is a 200 × 24 matrix, in which each row represents the integral value of the NMR spectrum of each patient, and Y represents the patient's condition where 1 indicates burn and 0 indicates health. A PLS model will try to find the multidimensional direction in the X space that explains the maximum multidimensional variance direction in the Y space; that is, it will try to find the spectrum variables in X that can explain the result of burn or health in Y. PLS is particularly suited when the matrix of predictors has more variables than observations.

In order to avoid excessive classification, we further adopted cross-validation (CV) to evaluate the stability of the model. We addressed the validation by cutting a single observation from the original sample as the validation data and the remaining observations as the training data. Each observation in the sample is used once as the validation data in turn. The Q^2^ value represents the percentage of the variation in the dataset predicted by the model according to CV, that is, the Q^2^ value represents the discriminating ability of the PPM of a particular segment. The formula for Q^2^ is as follows:


Here PRESS is the predictive residual sum of squares, and SSY is the sum of squares of the Y matrix. These measures can be equivalently expressed as standard error of prediction (SDEP or SEP), or standard error of CV (SECV).

Here, we get R^2^ = 0.87, Q^2^ = 0.76, and SECV = 0.201 with SD = 0.225

SECV is closed to the SD of the X matrix. It can be interpreted as the SEVC is closed to the NMR spectrum noise, so the stability of our model is acceptable.

Supporting vector machines (SVMs) have been successfully applied to various scientific problems, particularly in high-dimensional data, and a SVM generally achieves classification performance superior to that of many older methods. We employed a kernel function from quadratic, polynomial kernel, Gaussian Radial Basis, and multilayer perceptron to classify PLS scores.

#### Establishing metabolome and gene function analysis

The HMDB was used to identify key metabolites related to enzymes and upstream genes. In order to determine the common functions of these metabolites, we used the Gene Ontology terminology (GO) system to analyze the enrichment condition of above selected enzymes (and the corresponding genes). All GO analysis was conducted using the G: profiler website [16]. According to the website, the core algorithm in the program is the widely applied hypergeometric distribution for significance of the estimation principle for functional genomics of enrichment and analysis.

#### Computing platform and tools

All computation processes were conducted using a high-performance computing platform (HPC, CPU XEON E7-8870 2.4G 6.4GT/s30M10C *4, GPU TESLA K20 5GB GENERIC, 512GB DDR3 1333MHz R-ECC; Environment: Unbutu12.04) of the Metabolomics and Multidisciplinary Laboratory of Sichuan Academy of Medical Sciences with computing software Matlab 2012b.

## Results

### Patients

Of the 21 participants initially recruited, none withdrew consent. The patients’ average age was 43.2 ± 10.7 years, and they were admitted within 24 hours after injury to the participating hospitals. The patients’ average percentage of TBSA was 77 ± 12% (IQR 55 to 97%) and percentage of full thickness surface area (FTSA) was 45 ± 24% (IQR 5 to 95%). All patients were immediately given antishock fluid resuscitation upon admission, and all interventions were in accordance with the burn treatment guidelines issued by the Chinese Burns Medical Association. Four patients with severe burns died of multiple organ failure and sepsis. The overall mortality rate during the study period was 19%.

### Clinical assessments

All of the variables followed a normal distribution. Table 1 demonstrates that the two groups were comparable for basic demographic data. The subjects were similar in age and body weight. However, there were significant differences between the groups in the percentage of TBSA of burns, breathing rate, blood pressure (BP), pulse, and temperature.

**Table 1:** **Comparison of clinical data between severe burn patients and controls/**

Variables	Control (n = 3)	Case (n = 21)	***P*** value
Age (yr)	45.1 ± 7.4	43.2 ± 10.7	*>0.05*
Sex, male (female)	2 (1)	16 (5)	*>0.05*
Weight (kg)	61 ± 4	64 ± 13	*>0.05*
TBSA burn (%)	0	77 ± 12	
Second-degree burn (%)	0	37 ± 23	
Third-degree burn (%)	0	45 ± 24	
Breathing rate (times/min)	18.3 ± 2.2	22.2 ± 2.1	*<0.05*
BP (mmHg)	108 ± 14/75 ± 9	123 ± 18/82 ± 11	*<0.01*
Pulse (times/min)	78 ± 12	113 ± 18	*<0.01*
T (°C)	36 ± 0.3	36.8 ± 0.9	*<0.05*

Data are expressed as n (%) or mean ± standard deviation. TBSA, total body surface area; BP, blood pressure; T, temperature.

### Plasma metabolome after severe burn

**Figure 1:**
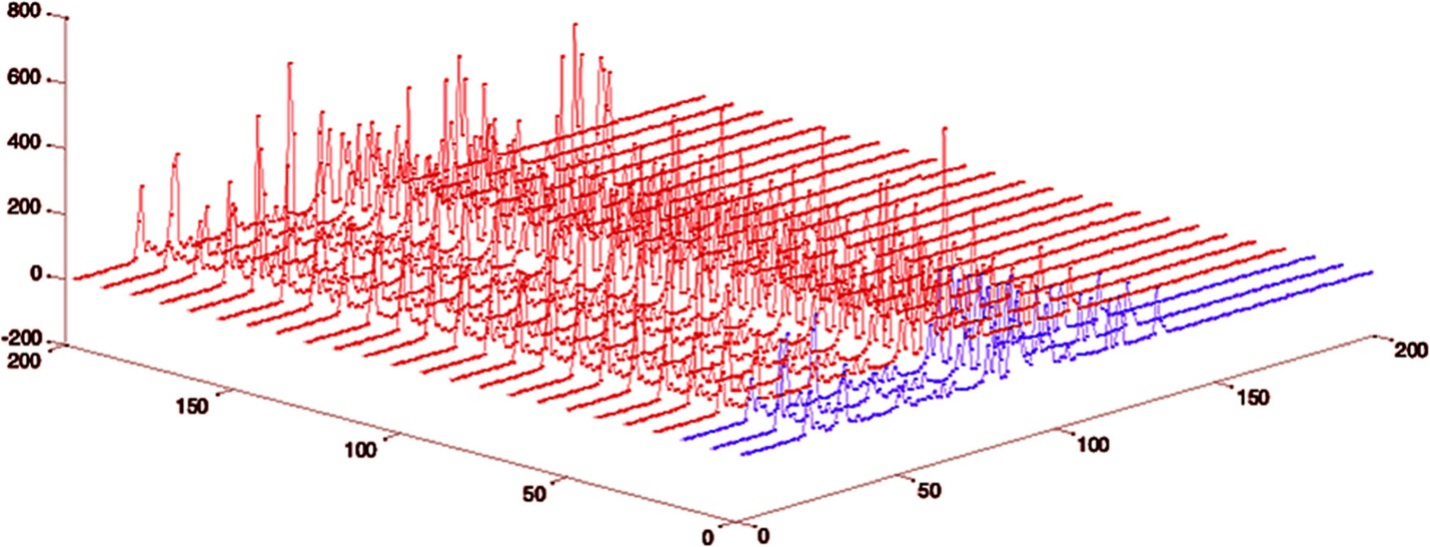
**Comparison of** **nuclear magnetic resonance** **(NMR) spectra from healthy controls and burn patients.** The blue line is the ^1^H-NMR plasma spectrum of healthy controls, and the red line is the NMR plasma spectrum of burn patients.

Typical 600.13-MHz NMR spectra demonstrated resonances arising from metabolites including glucose, histidine, and creatine. The differences between spectra from the severe burn patients and those from the controls were obvious on visual inspection (Figure 1), which demonstrates that there were significant alterations in the plasma metabolite profiles.

**Figure 2:**
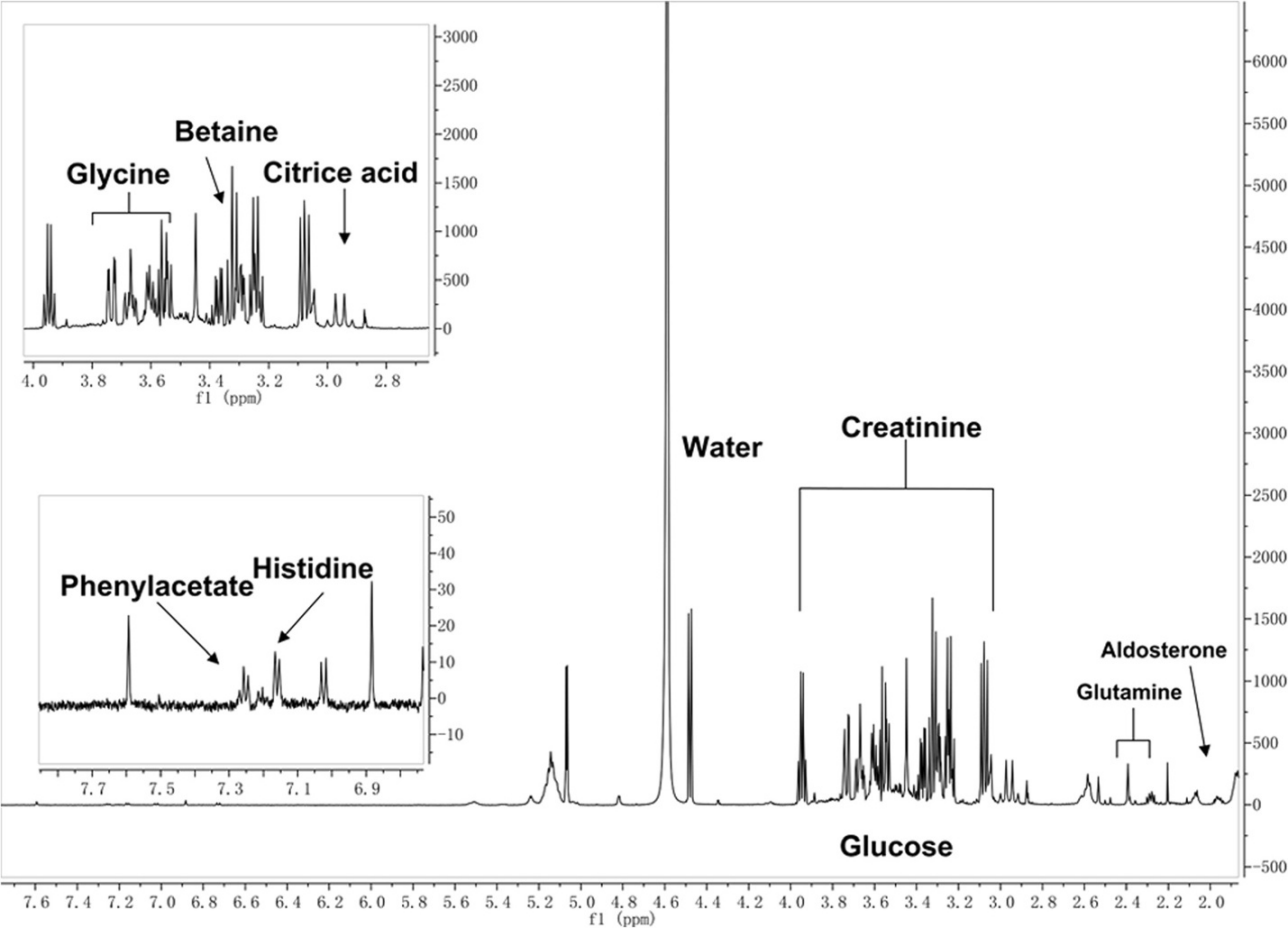
**A high-resolution** ^**1**^ **H-** **nuclear magnetic resonance** **spectrum of plasma demonstrating spectral assignments.** Only the major metabolites are labeled.

**Figure 3:**
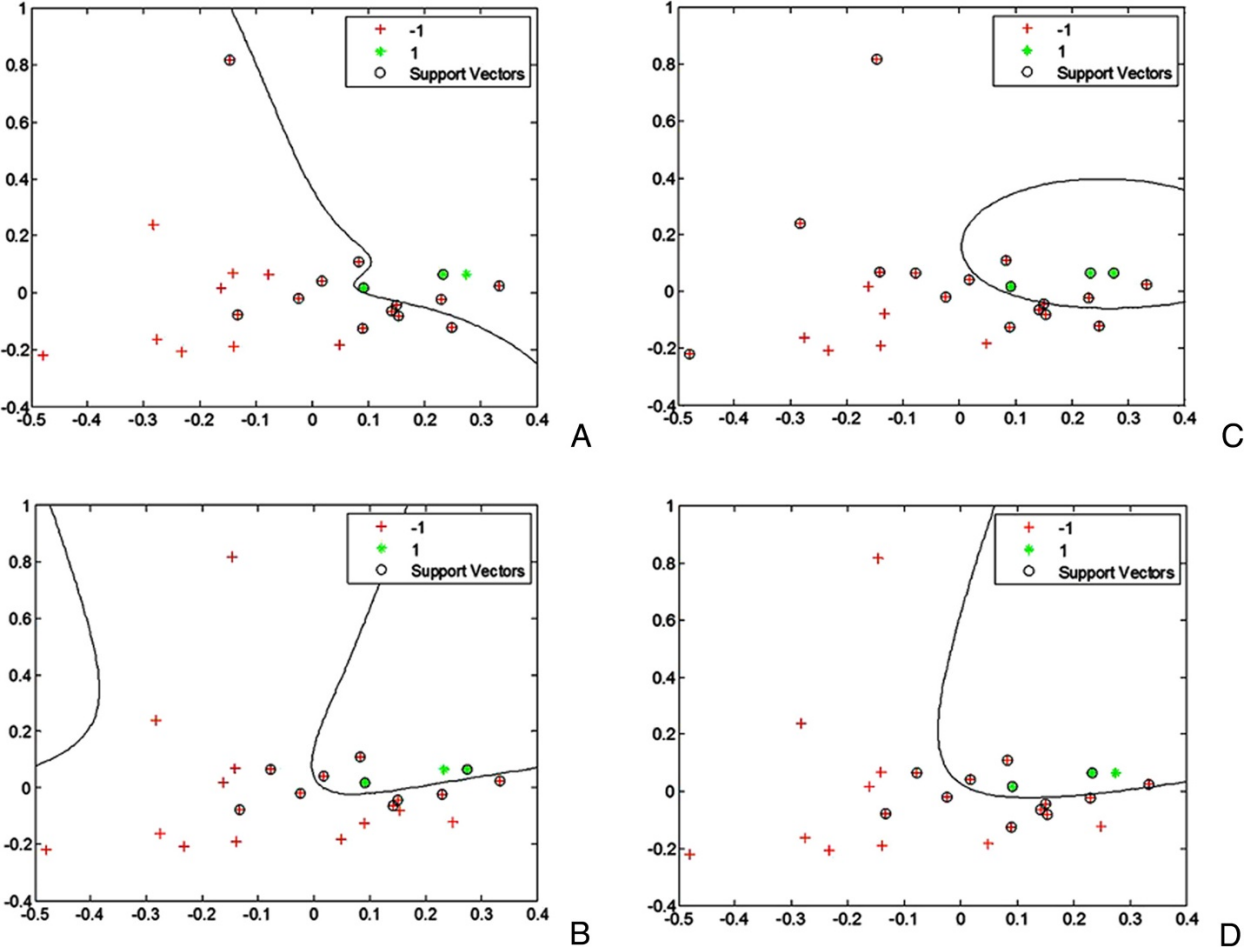
**Automatic separation of the sample score plot by support vector machine (SVM).** The kernel function of the SVM is quadratic **(A)**, polynomial kernel **(B)**, Gaussian Radial Basis **(C)**, and multilayer perceptron **(D)**. The black line is the separating line between the burn (+) and healthy (*) samples.

The variable importance in the projection (VIP) represents the value of each predictor in fitting the PLS model for both predictors and responses, and we used the method that was developed by Chong and Jun [17] to calculate VIP scores. The VIP indicator can describe correlations between the variable (X) and response (Y). We used the VIP to identify metabolites correlated with severe burns and named these metabolites as the 'Eigen-metabolome’ of severe burns. We used a VIP score >1.5 as a threshold to obtain the determinant metabolites [18]. Then we used the HMDB to identify 12 metabolites that are catalyzed by 103 enzymes (Tables 2 and 3 and Figures 2 and 3). These 12 metabolites represent the major metabolic changes that occur after severe burn and can be used as the Eigen-metabolome.

**Table 2:** **Summary of Eigen-metabolome: metabolites related to severe burn**

HMDB metabolite	HMDB enzyme - gene symbol						
3-Methylhistidine	CNDP1	PRMT3					
1,3-Diaminopropane	AOC2	AMD1	AOC3	SMS	DHPS	ABP1	ODC1
2-Hydroxybutyric acid	DLD	LDHB	LDHAL6B	LDHC	LDHAL6A	TDH	
2-Methoxyestrone	UGT1A1	UGT2B11	UGT2A3	UGT2B10	UGT1A5	SHBG	UGT1A8
	UGT2B15	UGT1A7	UGT2B7	UGT2B4	UGT2B28	UGT1A3	UGT1A6
	UGT2B17	COMT	UGT1A4	UGT2A1	UGT1A9	UGT1A10	
Deoxycorticosterone	HSD3B1	P450-CYP21B	CYP11B2	NR3C2			
	HSD3B2	CYP11B1	CYP21A2	NR3C1			
Alpha ketoisovaleric acid	-						
Iodotyrosine	TPO						
Biotin	PCCA	SLC5A6	PC	PCCB	ACACA	MCCC1	
	MCCC2	DKFZp686B20267	HLCS	ACACB	BTD		
7-Dehydrocholesterol	SC5DL	HMGCS2	SCP2	DHCR24	CYP11A1	DHCR7	
Aldosterone	MLPH	SGK1	NR3C2	NPPB	CYP11B1		
	FN1	CTGF	NR3C1	AKR1D1	ADM		
	CYP11B2	AGTR1	PTGER4	EGFR	PRKD1		
Dihydrobiopterin	TYR	TH	NOS3	DHFR	PCBD1		
	TPH1	QDPR	SPR	NOS1			
Butyric acid	HDAC1	TNF	PPARG	ACSM5	HDAC4	ACSM2A	
	HDAC5	HDAC2	ACSM4	ACSM1	SLC16A1	HDAC3	
	ACSM2B	ACSM6	CASP3	ACSM3	HDAC9		

Twelve characteristic metabolites found in nuclear magnetic resonance (NMR) metabolic spectra. They are closely related to substance metabolism, skeletal muscle and fat catabolism, or viscus functional disorder after severe burn injury. HMDB, Human Metabolome Database.

**Table 3:** **Biological processes associated with the 12 characteristic metabolites**

***P*** value	T	Q	Q&T	Q&T/Q	Q&T/T	Term ID	Main function	Gene dosage	Functional description
7.18E-05	42	89	6	0.067	0.143	GO:0006476	BP	7	Protein deacetylation
1.69E-02	1274	87	18	0.207	0.014	GO:0009611	BP	80	Response to wounding
1.67E-04	30	72	5	0.069	0.167	GO:0042312	BP	31	Regulation of vasodilation
3.43E-03	28	60	4	0.067	0.143	GO:0055078	BP	77	Sodium ion homeostasis
1.22E-02	2	45	2	0.044	1	GO:2001295	BP	41	Malonyl-coenzyme A biosynthetic process
1.80E-02	32	79	4	0.051	0.125	GO:0048662	BP	52	Negative regulation of smooth muscle cell proliferation
4.14E-02	89	72	5	0.069	0.056	GO:0046209	BP	45	Nitric oxide metabolic process
4.93E-33	944	88	45	0.511	0.048	GO:0019752	BP	2	Carboxylic acid metabolic process
3.23E-02	41	71	4	0.056	0.098	GO:0050999	BP	59	Regulation of nitric oxide synthase activity
2.75E-02	83	71	5	0.07	0.06	GO:0051341	BP	53	Regulation of oxidoreductase activity
3.31E-02	72	85	5	0.059	0.069	GO:0006096	BP	51	Glycolysis
4.64E-15	7548	88	73	0.83	0.01	GO:0044444	CC	33	Cytoplasmic part
1.14E-02	3100	89	31	0.348	0.01	GO:0031974	CC	74	Membrane-enclosed lumen
5.29E-05	40	89	6	0.067	0.15	GO:0000118	CC	4	Histone deacetylase complex
2.54E-29	33	31	14	0.452	0.424	GO:0015020	MF	21	Glucuronosyltransferase activity
7.80E-04	185	72	8	0.111	0.043	GO:0005506	MF	27	Iron ion binding
1.94E-02	3	33	2	0.061	0.667	GO:0004769	MF	81	Steroid delta-isomerase activity
1.05E-14	58	79	12	0.152	0.207	GO:0033293	MF	60	Monocarboxylic acid binding
7.06E-09	11	89	6	0.067	0.545	GO:0031078	MF	62	Histone deacetylase activity (H3-K14-specific)
3.84E-02	149	72	6	0.083	0.04	GO:0020037	MF	54	Heme binding
4.77E-04	1195	89	20	0.225	0.017	GO:0019899	MF	9	Enzyme binding
3.76E-06	614	68	15	0.221	0.024	GO:0042803	MF	38	Protein homodimerization activity

Biological processes associated with the 12 characteristic metabolites.

We were interested to use the Eigen-metabolome to establish a quantitative burn evaluation model. We employed a PLS regression model to establish a linear prediction model.

Then we obtained the discriminant equations for the relationship between plasma metabolites and injury severity:


where x represents the ppm value from the NMR spectra, and a_ij_ represents the loadings. Finally, we obtained an injury severity discriminant model based on a SVM. We found that SVM equations successfully distinguished severe burn patients and healthy control individuals.

## Discussion

In this study, we found that the metabolomics fingerprint of severe burn patients was altered significantly. Twelve small molecular metabolites (Table 2) make up an Eigen-metabolome that distinguishes severe burn patients from healthy controls. Hence, this Eigen-metabolome comprises a set of biomarkers that can be used to monitor the metabolism disturbances after severe burn injury. To the best of our knowledge, this is the first study on human metabolomics fingerprinting after severe burn. In addition, we identified several interesting findings regarding metabolic pathway regulatory changes in metabolomics fingerprinting.

One very interesting metabolite included in the severe burn Eigen-metabolome was α-ketoisovaleric acid, an intermediate metabolite of valine, that is regarded as a marker of mitochondrial damage [19, 20]. Valine can be converted to α-ketoisovaleric acid through a deamination reaction by branched-chain amino acid aminotransferase, and it is carried from the cytosol to mitochondria through the mitochondrial membrane transporter, where it is further converted to succinyl-coenzyme A by acyl-coenzyme A dehydrogenase and participates in the tricarboxylic acid cycle. Ketoisovaleric acid accumulates with disorder of the mitochondrial membrane transport system [21, 22]. In this study, we found that the plasma ketoisovaleric acid level significantly increased after burn injury, indicating cell membrane damage and mitochondrial transport dysfunction in the early stage of burn injury. Although our and others’ previous studies have demonstrated that mitochondrial dysfunction can be found one day post burn, those data were derived from animal tissues [23–26]. To date, there is no clinically available tool to monitor mitochondrial function. The present study indicates that through ^1^H-NMR metabolic fingerprinting, α-ketoisovaleric acid can be used clinically as a new biomarker of mitochondrial dysfunction.

Researchers have found that the 3-methylhistidine (3-MH) level reflects skeleton muscle degeneration after burn. A clinical study indicated that urine 3-MH is significantly increased in burned children [27]. It is seen as an important marker of catabolic metabolism. The traditional way to examine skeleton muscle decomposition is to detect 3-MH by high performance liquid chromatography (HPLC) and/or mass spectrometry (MS), but these methods are expensive and time-consuming. Because of the complexity of plasma contents, previous studies all used urine samples. The results from urine testing are not as accurate as those from plasma testing, which limits the use of 3-MH as a clinical marker. Our study found that as a high-throughput method, the ^1^H-NMR metabolome accurately and timely duplicated results that only could be examined in complicated laboratory studies previously. Because of its cost-effectiveness, ^1^H-NMR metabolome fingerprinting could be used as a sensitive monitoring tool for skeleton catabolism after severe burn.

After severe burn, the stress process is followed and represented by the release of a large amount of stress-related hormones and cytokines. This stress process leads to mal-metabolism of carbohydrates via insulin resistance and hyperglycemia [28]. For a very long time, researchers and clinical practitioners have considered hyperglycemia post severe burn as a type of stress-related phenomena and quite different from diabetes. They believe that this type of hyperglycemia is not related to ketonemia during the early stage of burn and that ketonemia only occurs when patients are suffering from sepsis [29]. Our study explored a different scenario: β-hydroxybutyric acid was increased in the metabolome of patients in the early stage post burn. Considering that β-hydroxybutyric acid is the major component of ketones (75% of ketones), the increasing level of β-hydroxybutyric acid indicated that ketogenic metabolism is enhanced by fatty acid decomposition in liver.

Through the above analysis of key metabolites from the Eigen-metabolome, we conclude that mitochondrial function and carbohydrate, protein, and fatty acid metabolism are significantly changed during the early stage of severe burn. The core cause of these types of changes is the decomposition of skeleton muscle and fat tissue to provide substrates for gluconeogenesis and ketogenic metabolism. The outcome of this metabolic adjustment is to fulfill the energy needs of brain and myocardial cells under stress conditions. All of this metabolic information could be obtained from a ^1^H-NMR spectrum, which indicates that ^1^H-NMR-based metabolomics fingerprinting can be used as a sensitive monitoring tool for severe burn patients. This also offers a new approach to understanding the complicated metabolic changes after severe illnesses and injuries such as burns.

Upon analysis of the 12 metabolites of the metabolome from severe burn patients, we found that they are catalyzed by 103 enzymes that mainly participate in biological processes including protein acetylation, wound healing, and dilation of blood vessels. From the cellular perspective, these enzymes are closely related to the deacetylation of histones, which means remodeling of chromatin and affects the dynamics of chromatin folding during gene transcription [30]. Our results showed that levels of the histone deaceylase (HDAC) components (HDAC1-HDAC5, and HDAC9) were elevated significantly and the affinity between histones and DNA was increased, eventually leading to gene transcription repression [30, 31]. However, the histone acetyltransferases (HATs), which reduce histone and DNA affinity and promote transcription, were not obviously changed. These results indicate that protein transcription and synthesis were inhibited and anabolism was restrained during the early stage of burn injury [32, 33].

The present study indicates that after burn injury, the alterations of metabolism networks and patterns can be detected by a metabolomics techniques based on ^1^H-NMR. On one hand, we found that 12 metabolites make up a set of biomarkers that can be used to monitor the severity of burns. Although the clinical standards for evaluating the severity of burns are well established and it is not difficult to distinguish severe and moderate burns, differences in the complex network of metabolism are not so easily understood. Our work used the disturbances in the metabolic fingerprints of 1H-NMR spectra to provide a quantitative method to describe the metabolic network disturbances after severe sepsis. It provides a systems biological approach to understand the relationships between metabolic network disturbances and the occurrence of morbidities (sepsis, multiple organ dysfunction syndrome, and so on) after severe burn.

In addition to establishing the Eigen-metabolomeour results demonstrate that α-ketoisovaleric acid can be used as a novel biomarker of mitochondrial dysfunction in the clinical setting. However, further analysis of the spectra of metabolites can go deeper and wider along the route of small molecular metabolite-enzyme-functional genomics, which provides innovative ideas for exploring pathophysiologic conditions, enhancing research efforts, and improving future treatment protocols. Finally, our study provides a novel approach for a clinical monitoring system with high sensitivity and accuracy in the future.

## Conclusions

To summary, we demonstrate that ^1^H-NMR spectra can be used to establish Eigen-metabolome of severe burn patients. A set of biomarkers such as α-ketoisovaleric acid, 3-methylhistidine, and β-hydroxybutyric acid can characterize metabolic disturbances after severe burn. Our work also provides a systems approach to biomedicine that enable future researchers to integrate information from clinical settings, metabolomics and mathematical modeling to develop a new diagnostic monitoring tool for severe burn patients.

## Key messages

- NMR spectra of plasma samples showed significant differences between burn patients and healthy individuals.
- Using metabolomics techniques, we identified an Eigen-metabolome that consists of 12 metabolites, which are regulated by 103 enzymes in the global metabolic network.
- α-ketoisovaleric acid, 3-methylhistidine, and β-hydroxybutyric acid were the most important biomarkers that were significantly increased during the early stage of burn injury.
- Our results also show that the histone deacetylases, which are protein transcription suppressors, were remarkably increased and indicated that protein transcription was inhibited and anabolism restrained during the early stage of burn injury.

## Abbreviations

BP: blood pressure
CV: cross-validation
FTSA: full thickness surface area
HAT: histone acetyltransferase
HDAC: histone deacetylase
HPLC: high-pressure/performance liquid chromatography
IQR: interquartile range
MH: methylhistamine
MS: mass spectrometry
NMR: nuclear magnetic resonance
PCA: principal component analysis
PLS: partial least squares
PLS-DA: partial least squares discriminant analysis
SD: standard deviation
SDEP: standard error of prediction
SECV: standard error CV
SMV: supporting vector machine
TBSA: total body surface area
VIP: variable importance for projection.
